# Preparation of Anisotropic Aerogels with Pristine Graphene by Heat Flow and Study of Their Effects on Heat Transfer in Paraffin

**DOI:** 10.3390/nano9111622

**Published:** 2019-11-15

**Authors:** Jinhui Huang, Buning Zhang, Ming He, Xue Huang, Guoqiang Yin, Yingde Cui

**Affiliations:** 1 School of Materials Science and Engineering, Northwestern Polytechnical University, Xi’an 710072, China; 2 Guangzhou Key Laboratory for Efficient Utilization of Agricultural Chemicals, Zhongkai University of Agriculture and Engineering, Guangzhou 510225, Chinaheming1026@163.com (M.H.); huangxue0206@126.com (X.H.); 3 Guangzhou Vocational College of Science and Technology, Guangzhou 510550, China

**Keywords:** anisotropic aerogels, paraffin, phase-change energy storage

## Abstract

In this study, anisotropic graphene/graphene oxide (GO) aerogels (AGAs) were obtained by freeze-drying after direct participation of pristine graphene in the self-assembly of anisotropic gel by the heat flow method. After vacuum microwave treatment, the physical, chemical and structural characteristics of the AGAs were investigated. The results show that AGAs, in which the internal graphene sheets are parallel to the heat flow direction, are successfully prepared. After microwave treatment, the amount of oxygen and nitrogen reduces significantly and the sp2 domain increases. However, at the same time, many fragments and holes are generated in the graphene sheets. The effects of AGAs on the phase transition of paraffin is studied, and the results show that the melting enthalpy, solidification enthalpy and initial melting temperature of AGA/paraffin composites decreases as the GO content in the AGAs increases, whereas the melting range, solidifying range and subcooling degree increases. The highest axial thermal conductivity of the AGA/paraffin composite is 1.45 W/(mK), and the thermal conductivity enhancement efficiency is 884% (AGA content was 0.53 vol %). Compared with previously investigated, similar AGA/paraffin composites, the aerogels fabricated in this study have the obvious advantages of a simple fabrication process, a low cost and a high thermal conductivity enhancement efficiency. These aerogels possess the potential for application in phase-change energy storage (PES), thermal energy management and other fields.

## 1. Introduction

Despite the discovery and development of new energy sources and improvements in the utilization efficiency of existing energy sources, mankind’s demand for energy has neither waned nor ceased. Phase-change energy storage (PES), as a new form of energy storage, can improve the rate of the utilization of existing energy, and has developed rapidly in recent years. Many important research achievements are made, from the basic theory to product development. However, there are still many problems, such as low thermal conductivity and leakage or shape instability during the phase change process [1], which need to be resolved for practical application. 

Admittedly, many thermal conductive types of filler, such as graphite [2,3,4], expanded graphite [5,6,7], carbon nanotubes [8,9,10], metal nanoparticles [11,12,13] and metal foam [14,15,16], etc., are added to improve the thermal conductivity of phase change materials. But there are many accompanying problems, such as a serious reduction of phase change enthalpy per unit mass, the material inhomogeneity caused by fillers aggregation after multiple cycles, and serious leakage or shape instability during phase change (graphite, carbon nanotubes and metal nanoparticles, etc.). Another option, such as the metal foam, has very high thermal conductivity enhancement effect and shape stability, but it is easy to be corroded, and the interaction force with organic phase change materials is not strong, which leads to leakage in this phase change process. In addition, not all performance improvements can be achieved at the same time; for example, expanded graphite can improve heat conduction very well, but a large amount is needed to prevent the leakage of the phase change process, causing the phase change enthalpy decrease.

The emergence of graphene offers a practical solution to these problems. When graphene is incorporated into an aerogel as a thermal conductive filler, many outstanding characteristics can produce a marked effect, such as a large specific surface area of the aerogel, a large number of holes, a strong capillary effect and the good oleophilicity of graphene itself, and these not only solve the problem of the low thermal conductivity of the phase change materials, but they also solve the leakage problem and shape instability during the phase change process [17]. Owing to its extremely high thermal conductivity, the addition of graphene to phase change materials can greatly improve their thermal conductivity, thereby shorting the phase change process and improving the heat transfer efficiency [18,19,20,21,22]. Therefore, the application of graphene aerogel (GA) to PES has become a topic of interest, and the preparation of high-performance GA is the key to this application.

Currently, there are many methods to prepare GAs, such as the chemical vapor deposition (CVD) template method [23,24], three dimensional (3D) printing [25] and hydrothermal reduction [26,27,28]. Among them, those prepared by the CVD template method have better properties, can retain the properties of the original graphene better, and have fewer defects. However, the preparation process has low yields and involves high temperatures, numerous steps and complex procedures. Moreover, toxic and harmful substances are required during the production process, and are also generated as by-products thereof. This renders production and application at the industrial scale difficult. The hydrothermal reduction method involves the reduction of GO, which has the advantages of simplicity of process, low cost, high yield and ease of scalability and adjustability. Thus, it is highly suitable for industrial production. This method has been widely studied, but the aerogel prepared by this method has severe limitations when applied to PES. First, although perfect single-layer graphene has a thermal conductivity of up to 5300 W/(mK) [29], in GAs prepared by the oxidation-reduction method, the reduced graphene oxide (rGO) sheets break and contain oxidation defects, vacancy defects and doping defects, which seriously affect the thermal conductivity of graphene. For example, the thermal conductivity of the oxidized graphene decreases to 0.14–2.87 W/(mK) [30], and the thermal conductivity of the GA decreases to 0.023 W/(mK) after doping with nitrogen atoms [31]. Second, graphene sheets randomly undergo self-crosslinking to assemble aerogels. However, owing to the large number of contact points and the low contact area between graphene sheets in an aerogel, the thermal conduction pathway is long and tortuous. This causes a significant increase in the thermal resistance of the contacts and serious phonon scattering, resulting in a sharp decline in thermal conductivity [32]. To overcome these problems, many researchers have repaired rGO at high temperatures. For example, Li et al. [33] quenched and repaired rGO at 2800 °C, and the properties of the resulting aerogel were very close to the properties of pristine graphene, and the thermal conductivity enhancement efficiency of the aerogel was as high as 5890%. In an attempt to reduce contact thermal resistance, Peng et al. [19] prepared anisotropic GA by the ice crystal template method. After adsorption of paraffin, the thermal conductivity reaches 8.87 W/(mK). Li et al. [34] prepared anisotropic GA by in-situ curing of ordered GO liquid crystals with gaseous hydrogen chloride (HCl). After adsorption of paraffin, its thermal conductivity reaches 2.99 W/(mK). However, the high-temperature repair method requires argon gas protection, which increases the cost and energy consumption and reduces the efficiency. 

The ice crystal template method is complex and difficult to control. However, it is difficult to prepare anisotropic pristine GAs using conventional methods, because graphene dissolves in water with difficulty and aggregates easily.

Therefore, an AGA containing pristine graphene was prepared by the heat flow method. After subsequent microwave treatment in vacuum and paraffin adsorption, an AGA/paraffin composite phase change material with a high thermal conductivity was prepared. Pristine graphene was directly involved in the aerogel self-assembly, which eliminates the negative effects on the thermal conductivity of defect generation and atom doping in the redox process. Moreover, the role of “heat flow” makes graphene orientational, which facilitates closer internal crosslinking of the AGAs, thus increasing the crosslinking area, reducing the contact thermal resistance, smoothening the thermal conductive pathways, and further improving the thermal conductivity performance.

## 2. Experimental

### 2.1. Preparation of AGAs

#### 2.1.1. Preparation of Graphene Dispersion

GO was prepared using an improved Hummer’s [35] Method, as detailed in previous work [36].

**Figure 1:**
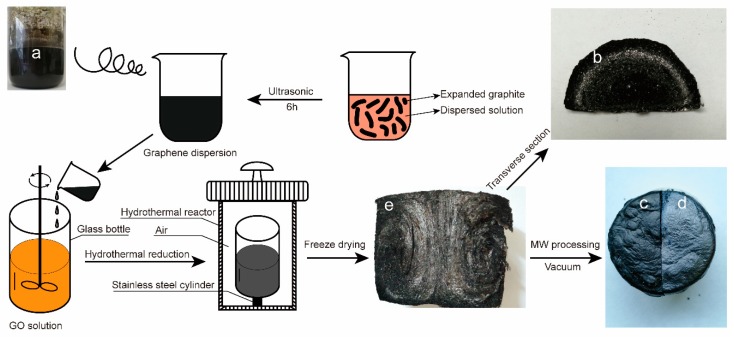
Schematic diagram of anisotropic graphene/graphene oxide (GO) aerogels (AGAs) preparation steps. A digital image of the graphene dispersion (**a**) and a digital image of the AGA’s transverse section (**b**); a digital image of the AGA before (**c**) and after microwave treatment (**d**); a digital image of the AGA’s longitudinal section (**e**).

GO (1.0 g) was added to 200 mL, 70% *v*/*v* ethanol solution and stirred under ultrasonication until it dissolved. Then, 1.0 g of expanded graphite (EG) was added and stirred under ultrasonication for 6 h to obtain a graphene dispersion solution. As shown in Figure 1a, the solution was still a uniform solution after 48 h, and no stratification was observed.

#### 2.1.2. Preparation of AGA

As shown in Figure 1, 0.175 g of GO was added to a glass bottle containing 45 mL of deionized water. After stirring and dissolution, 5 mL of the graphene dispersion was added to the glass bottle while stirring. Finally, 50 mL of the GO solution with a concentration of 4 mg/mL was obtained. Then the glass bottle was placed into a hydrothermal kettle, and the hydrothermal kettle was placed into an oven. The oven was heated, from 30 °C to 150 °C within 8 h, and kept for 1 h to obtain a wet gel. The gel was soaked in deionized water for 48 h, during which time the deionized water was replaced six times. After that, it was frozen for 3 h at −45 °C, freeze-dried for 48 h, and finally subjected to vacuum microwave treatment to obtain AGA. 

In this experiment, samples with different GO concentrations, different microwave treatment times and different amounts of added graphene dispersion were prepared. These were labeled as C*x*W*y*E*z*, with *x* representing the GO concentration (mg/mL), *y* representing the microwave treatment time (min) and *z* representing the amount of added graphene dispersion (mL). The prepared aerogel was immersed in the paraffin wax (Guangzhou chemical reagent factory, Guangzhou, China, #52, Melting point 50–52 °C, Product standards Q/SOCC 07), heated and melted, and absorption was conducted in vacuum for 30 min. It was then taken out for natural cooling to obtain AGA/paraffin composites, labeled as PC*x*W*y*E*z*. For example, C8W1E5 indicates that the concentration of GO solution is 8 mg/mL, and the amount of graphene dispersion was 5 mL when preparing the sample. After freeze-drying, the aerogel was microwaved for 1 min.

### 2.2. Performance and Structure Characterization

The morphologies of these AGAs were investigated using scanning electron microscopy (SEM) with a EVO18 microscope (Carl Zeiss, Germany). X-ray diffraction (XRD) patterns were measured with a D8 Advance (Brucker, Germany) diffractometer (Cu Kα radiation) at a generator voltage of 40 kV. Raman mappings were carried out with a microscope confocal Raman spectrometer (Horiba Jobin Yvon LabRAM HR800, Longjumeau, France) at an excitation wavelength of 633 nm. Thermal performance analysis of our samples were measured by a differential scanning calorimeter (DSC, Q2000, TA Instruments, New Castle, DE, USA) at a scanning rate of 10 °C min^−1^. Thermogravimetric analysis (TGA) curves were obtained with a TGA2 (Mettler Toledo, Columbus, OH, USA) at a heating rate of 10 °C min^−1^ in a nitrogen atmosphere. Elemental analysis was carried out by X-ray photoelectron spectroscopy (XPS, ESCALAB 250Xi, ThermoFisher Scientific, Waltham, MA, USA) with the following parameters: Monochrome Al Ka (*hv* = 1486.6 eV), power 150 *W*, 500 µm beam speckle. The binding energy was calibrated at C1s 284.8. Xpspeak41 software was used for peak fitting, with 20% Gaussian and 80% Lorentzian fixed and Shirley background mode adopted. Thermal imaging analysis was carried out by a thermal imager (Tis10, Fluke, USA). The thermal conductivity of these samples was measured by a thermal conductivity meter (hot-wire method, TC3100, XIATECH, China) at 25 °C. The fast response temperature chamber (GP/TH-50, SH Guangpin Test equipment manufacturing Co., Ltd., China) was used for investigating the thermal cycle stability of samples, which experienced several heating/cooling cycles between 10 °C and 70 °C, and the latent heat was measured by DSC after several cycles.

The enhancement efficiency in thermal conductivity of the paraffin composites can be calculated by

*η* = (*K* − *K_m_*)/(100 × *V* × *K_m_*) × 100%

where *η* is the thermal conductivity enhancement efficiency; *K_m_* and *K* are the thermal conductivities of paraffin and its composite, respectively; and *V* is the volume content of AGA in the composite.

## 3. Results and Discussion

**Figure 2:**
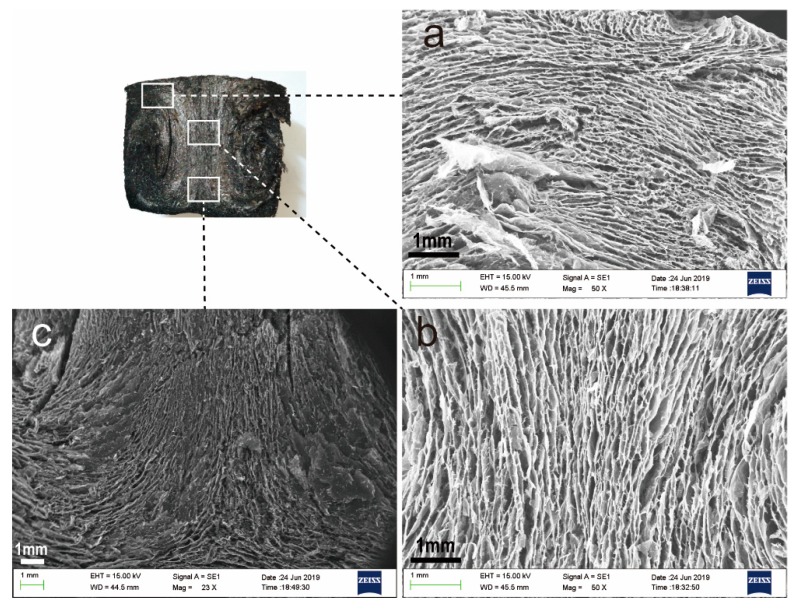
The scanning electron microscopy (SEM) images of AGA. (**a**–**c**) are electron microscope images of different positions in the longitudinal section of AGA.

**Figure 3:**
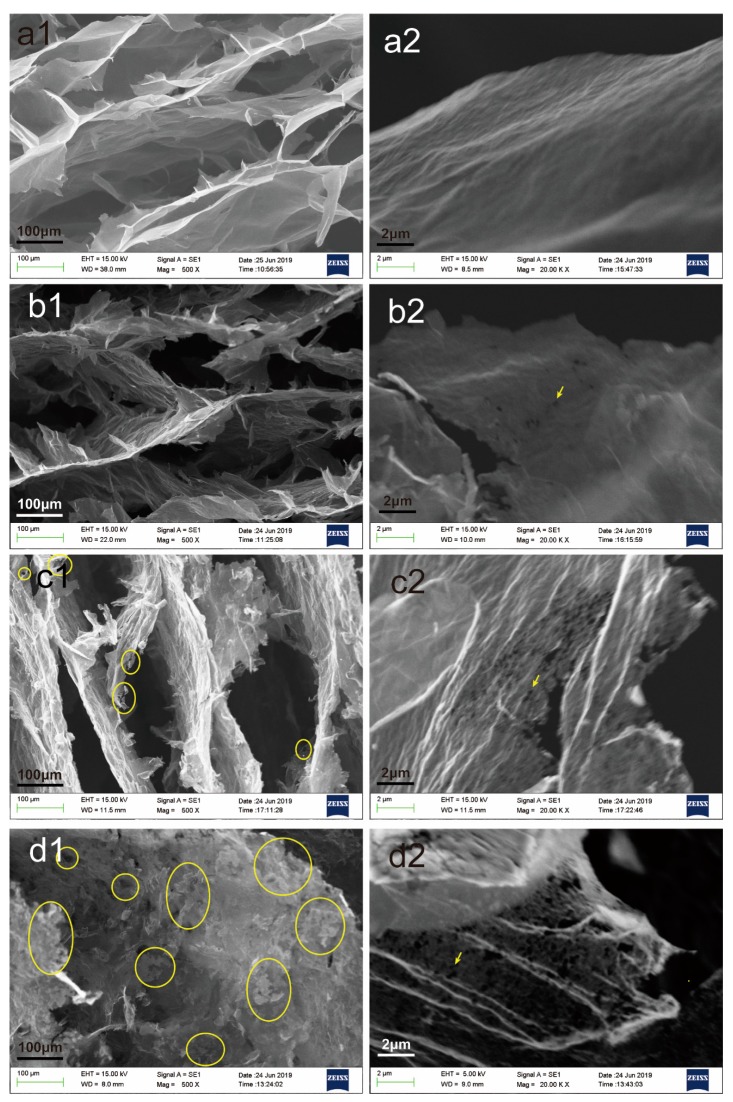
SEM images of graphene sheets in AGA with different microwave treatment times ((**a1**,**a2**) were unmicrowaved, (**b1**,**b2**) were microwaved for 1 min, (**c1**,**c2**) were microwaved for 5 min, (**d1**,**d2**) were microwaved for 20 min).

In this study, through directional heating, the temperature gradient was generated inside the solution to generate a heat flow, so that graphene sheets were oriented along the direction of heat flow and assembled into gels with a certain heat flow orientation. From Figure 1e, it can be seen that AGA has obvious heat flow directional characteristics. From the enlarged images of Figure 2 at different positions, it can also be seen that the graphene sheets are parallel to the direction of heat flow and that they change with the direction of heat flow, indicating that AGA with heat flow orientation was successfully prepared. From the cross-section of the AGA in Figure 1b, it is also evident that there is an inner ring, which is caused by heat flow turning, and here the graphene sheet is parallel to the bottom of the bottle, and the unique metallic luster of the graphite is thus observed. Figure 1c,d respectively show images of the AGA before and after vacuum microwave treatment. It can be clearly seen that AGA has an obvious metallic luster after microwave treatment. Figure 3 shows the morphology and characteristics of graphene sheets in AGAs after different microwave treatment times. As can be seen from the figure, as microwave processing time increases, the rough edges and fragments (shown in circles) in AGA increases, and the graphene sheet has many holes (shown in arrows). 

This may be due to the rapid increase in temperature during microwave heating and the rapid removal of adsorbed moisture and oxygen-containing and nitrogen-containing groups, resulting in the expansion of the stacked graphene sheets, which in turn results in the peeling of the edge graphene sheets. With the removal of the oxygen-containing and nitrogen-containing groups, the graphene sheet has a large number of pores at the positions where it was oxidized and doped.

**Figure 4:**
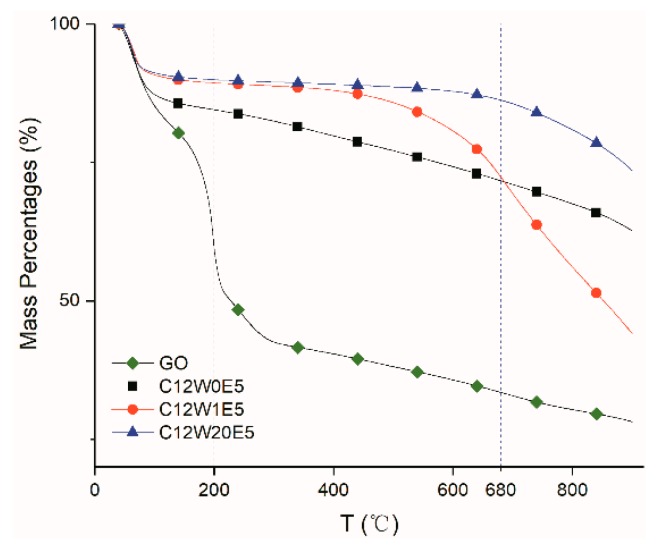
TGA curves of different samples.

The thermal stability of AGAs was analyzed by TGA. Figure 4 shows the weight loss curves of different aerogels. It can be seen from the curves that GO shows great weight loss at about 200 °C. But in those samples after hydrothermal reduction and microwave treatment, there is no weight loss at about 200 °C. This may be caused by hydrothermal reduction, which only reduces the oxygen-containing and nitrogen-containing groups that can decompose at about 200 °C (and below). As the temperature increases, the undecomposed groups are slowly decomposed. However, samples subjected to microwave treatment for 1 min or 20 min showweight loss at about 680 °C, with the former showing obvious weight loss, and the latter showing only a small amount of weight loss. 

This may be due to differences in microwave processing time, which leads to different temperatures inside the sample. Therefore, the types of oxygen-containing and nitrogen-containing groups decomposed are also different. In other words, microwave treatment for 1 min may decompose oxygen-containing and nitrogen-containing groups that can be decomposed below 680 °C, and leave only those that decompose at about 680 °C (and above). Therefore, samples subjected to microwave treatment for 1 min exhibit a large weight loss near 680 °C, whereas those subjected to microwave treatment for 20 min exhibit a small weight loss near 680 °C, because the temperature and time were sufficient to decompose groups containing oxygen or nitrogen. The differences in the types and contents of oxygen-containing and nitrogen-containing groups in different samples are also verified in the following XPS elemental analysis.

**Figure 5:**
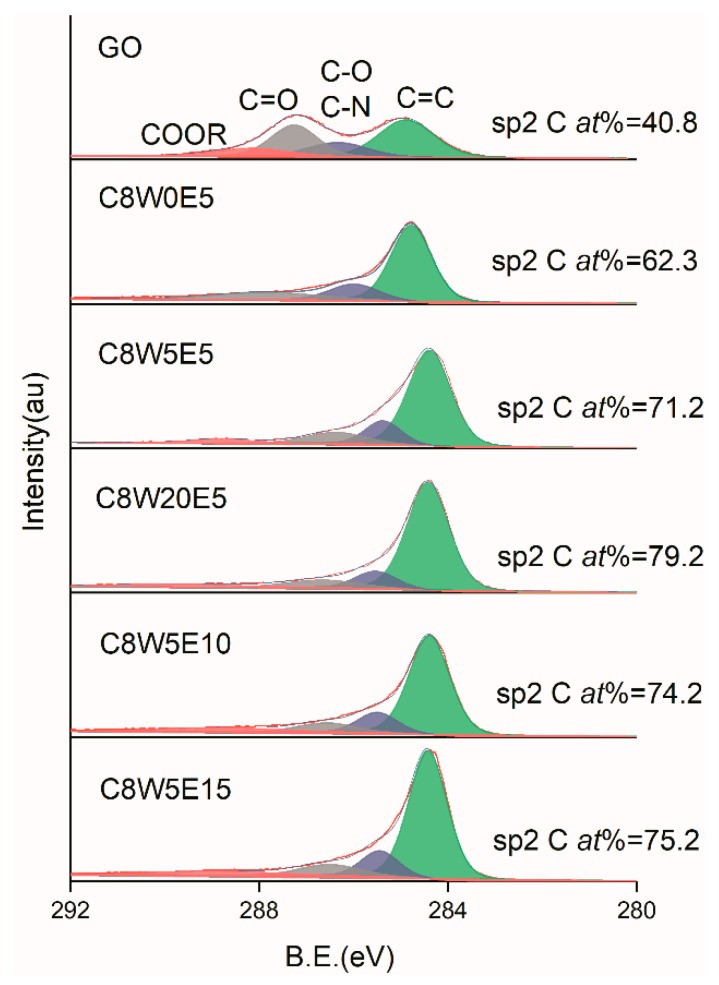
C1s X-ray photoelectron spectroscopy (XPS) fitting curves of different samples.

**Figure 6:**
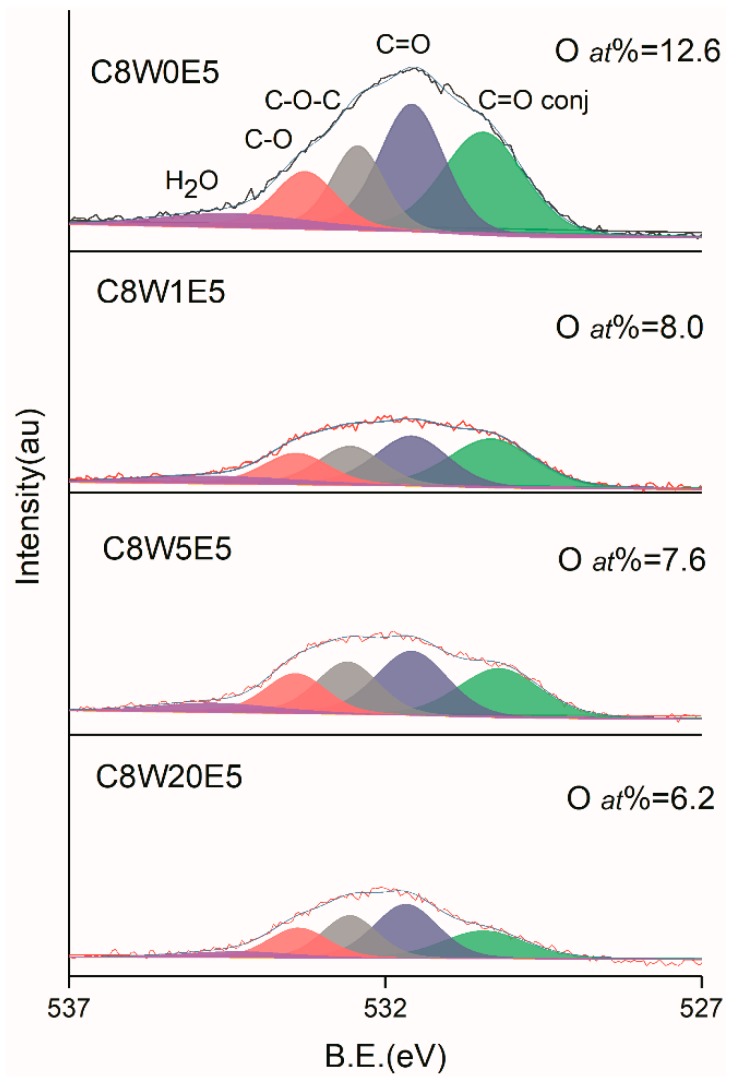
O1s XPS fitting curves of different samples.

**Figure 7:**
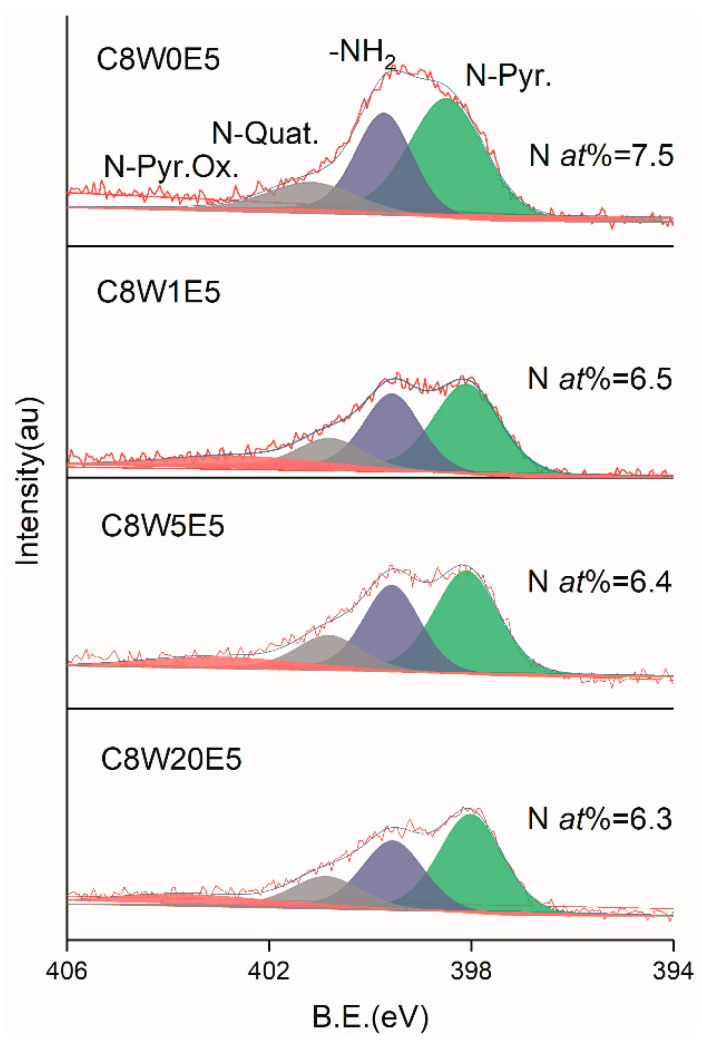
N1s XPS fitting curves of different samples.

Elemental analysis of the sample was carried out by XPS. Figure 5 shows the C1s analysis curves of GO and different AGAs. It can be clearly seen that after hydrothermal reduction and microwave treatment, the sp2 carbon atom content increases from 40.8% of GO to a maximum value of 79.2%. From the peak shape, the COOR and C=O groups show the most significant reduction. However, the C–O (C–N) content is not significantly reduced, and there is a red shift, indicating that C–O with large binding energy undergoes more decomposition, while C–N with small binding energy underwent less decomposition (verified in the following O1s and N1s analysis). Obviously, the content of sp2 carbon atom increases as the EG content increased. Furthermore, the sp2 carbon atom content also increases as the microwave treatment time increases, which indicates that the sp2 carbon atom content increases with the reduction of oxygen-containing and nitrogen-containing groups. Figure 6 is the O1s fitting curve. It can be seen from the figure that the oxygen content decreases gradually as microwave processing time increases. From the peak shape, various groups exhibit differing degrees of reduction. The most obvious reduction is exhibited by the C=O group, followed by the C–O and C–O–C groups. However, the degree of conjugate of C=O and C=C (C=O conj), [37] increases as the sp2 carbon atom content increases, and decreases as the C=O content decreases. After microwave treatment, the C=O group decreases greatly, resulting in a decrease of its relative content. Figure 7 is the fitting curve of N1s. It can be seen from the values in the figure that the decrease of nitrogen content is not obvious, but the relative change of –NH_2_ content is obvious from the change in the peak shape. This indicates that microwave treatment does not significantly reduce the nitrogen content, but may only decompose part of the –NH_2_ group and a small number of other groups. This verifies that C–O decomposition is more pronounced, and C–N decomposition is less so in C1s analysis.

**Figure 8:**
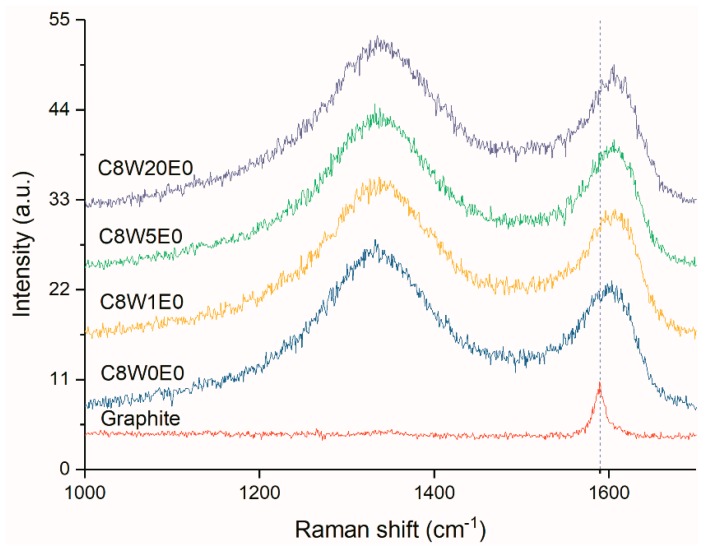
Raman curves of AGAs with different microwave processing times.

**Figure 9:**
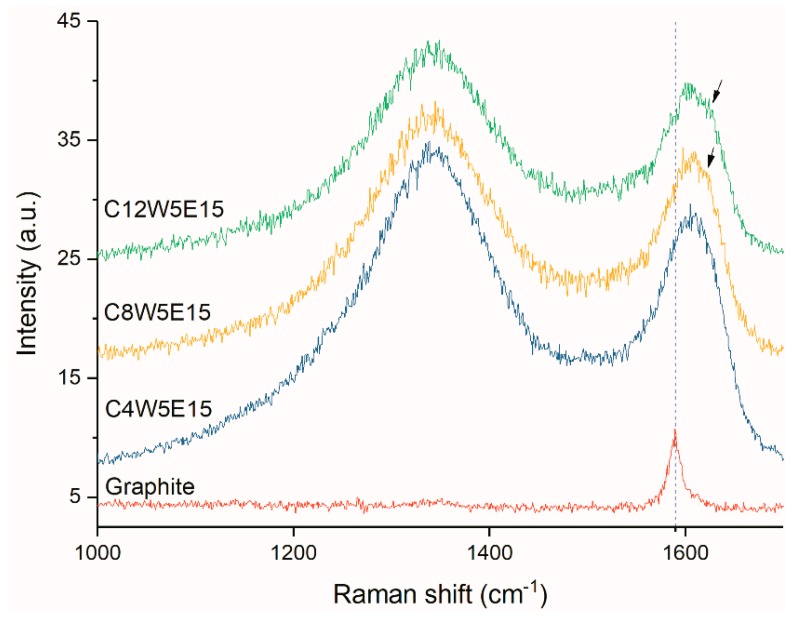
Raman curves of AGAs with different graphene oxide (GO) concentrations (The arrows indicate the blue shift).

The physical properties of the AGAs were analyzed by XRD and Raman. In the Raman spectrum, the peak around 1582 cm^−1^, commonly called as the G line, is caused by the Raman active E2g phonon (in-plane optical mode), which is close to the Γ point. The D line around 1350 cm^−1^ relative signal strength (compared to the G line) strongly depends upon the level of disorder in the graphitic material [38]. It can be seen from the Raman curves of AGAs with different microwave treatment times (Figure 8) that the G peak of AGAs is blue shifted. This is due to the fact that, after ultrasonic peeling and redox and microwave treatment, the graphene sheets in AGAs broke and contained many defects [39], resulting in a decrease in the average crystal size [40] and the presence of isolated double bonds [41]. Theoretically, with the removal of oxygen-containing groups, the sp2 carbon domain will increase, thereby decreasing I_D_ and increasing I_G_. However, it can be seen from the figure that I_D_/I_G_ remains unchanged. This may be due to the formation of many fragments and defects after hydrothermal reduction, resulting in a smaller average size of the sp2 domain, which leads to an increase in I_D_ [42], and the presence of more micropores in the graphene sheets after microwave treatment (it can be seen from Figure 3(c2,d2) that there were many micropores in the graphene sheet with the extension of the microwave processing time), thereby increasing defects. Moreover, the boundary effect also causes an increase in I_D_ [40]. As can be seen from the Raman curve of GA with different GO contents in Figure 9, with the increase in GO concentration, a shoulder peak (indicated by the black arrow) appears in the G peak. This peak can be attributed to the blue shift observed when EG concentration decreases with an increase in GO concentration (consistent with the analysis results in Figure 8).

**Figure 10:**
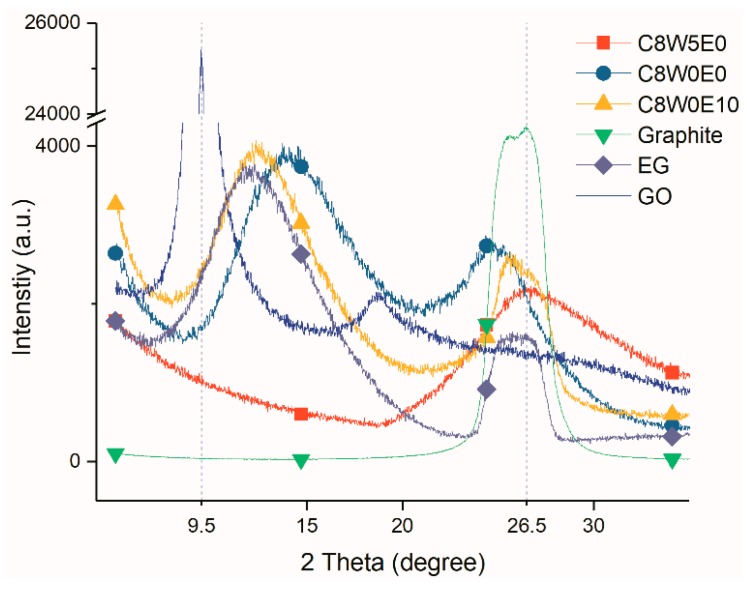
XRD curves of different samples.

The crystal structure of the AGAs was characterized by XRD. It can be seen from Figure 10 that EG has two diffraction peaks, one is at 26.5°, which is the characteristic diffraction peak of graphite, and the other is near 12°, which was caused by the larger spacing of some crystal planes after graphite was expanded. However, there are two peaks in expansible graphite, one at 26.5°, which is the characteristic diffraction peak of graphite, and the other is slightly lower than 26.5°, which is the result that the expansible graphite is intercalated with strong acid, resulting in an increase in the spacing of some layers. After GO is oxidized and intercalated [43], there is only one diffraction peak (9.5°), while the peak of 26.5° almost disappears, indicating that GO oxidation is relatively complete. After hydrothermal reduction, the interlayer spacing of graphene sheets in rGO decreases; therefore, the peak moves to the right, and diffraction peaks appear around 14°. However, due to incomplete reduction, oxygen-containing groups remain between the graphene sheets, resulting in larger interlayer spacing than that of perfect graphite [44]. Therefore, the characteristic peak of rGO appears at 25°. After microwave treatment, the diffraction peak of 14° completely disappears; however, a wider peak appears near 26°. 

**Figure 11:**
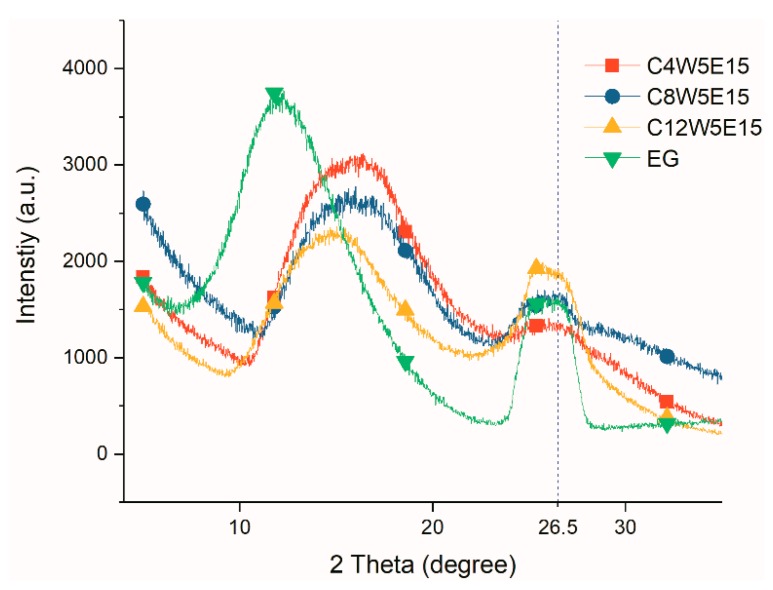
XRD curves of AGAs with different GO concentrations.

This is because after microwave treatment, the removal of a large number of oxygen- and nitrogen-containing groups [45] results in further narrowing of the rGO layer spacing. However, AGAs still contains small amounts of oxygen- and nitrogen-containing groups (as can be seen from XPS analysis), resulting in no diffraction peak at 26.5° (the peak of perfect graphite), but only infinitely close. After microwave treatment, rGO shows many fragments and holes (Figure 3(c1,d1,c2,d2)), as well as defects, resulting in smaller crystal size; therefore, the diffraction peak around 26° becomes very wide. After the addition of EG, because the AGAs contain both EG and rGO, their non-graphite characteristic diffraction peaks (near 12.8°) are between those of EG (12°) and rGO (14°). From the XRD curves of AGAs with different GO concentrations in Figure 11, it can be seen that, with the decrease in GO concentration and the increase in relative EG content, the peak strength of EG near 15° gradually increases (According to the analysis in Figure 10, after microwave treatment, rGO only shows a diffraction peak near 26°; hence, the peak here is the diffraction peak of EG). However, the intensity of the diffraction peak around 26.5° is mainly affected by the content of GO. The higher the concentration of GO, the higher is the intensity. Moreover, as analyzed in Figure 10, the peaks here become very wide.

**Figure 12:**
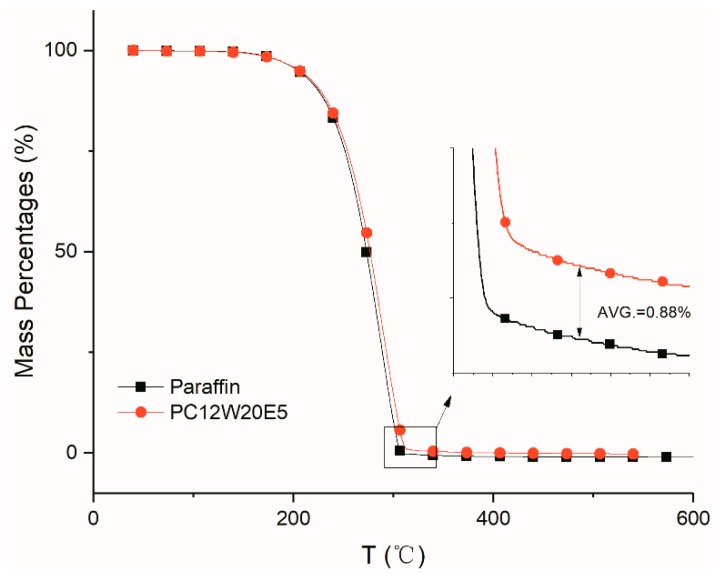
TGA curves of paraffin and PC12W20E5.

**Figure 13:**
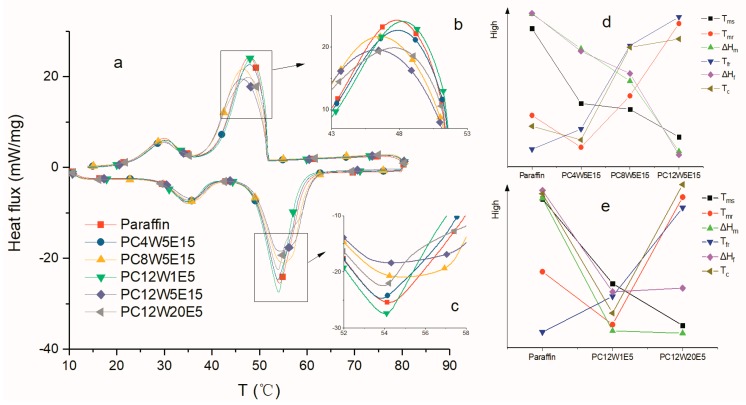
DSC curves of different AGA/paraffin composites ((**a**) was the complete curve, (**b**) and (**c**) were the local enlarged pictures of the melting and solidification curves respectively); Figure (**d**) compared the relevant thermal properties of samples with different GO concentrations and the same other conditions. Figure (**e**) compared the relevant thermal properties of samples with different microwave treatment times and the same other conditions. (T*_ms_*, the initial melting temperature; T*_mr_*, the melting range; ∆H*_m_*, melting enthalpy; T*_fr_*, solidifying range; ∆H*_f_*, solidification enthalpy; T*_c_*, subcooling degree. See Table 1 for specific values).

After the AGA adsorbed paraffin in vacuum, the thermal stability of the composites and the effect of AGA on the phase transition behavior of paraffin were studied. Figure 12 shows the thermal decomposition curve of paraffin and PC12W20E5. It can be seen that paraffin is completely decomposed when the temperature is around 300 °C, while the mass of PC12W20E5 is about 0.88%, which is the mass of AGA in PC12W20E5 after thermal decomposition at about 300 °C (Shown in Figure 12). Figure 13 shows DSC curves of different AGA/ paraffin composites. From the peak shapes and positions of Figure 13b,c, it can be seen that the addition of AGA has a significant impact upon the paraffin transition temperature, the phase transition process and the enthalpy of the composites. It can be seen from Figure 13d that the T*_ms_*, ∆H*_m_* and ∆H*_f_* of the composite material after the addition of AGA are lower than those of pure paraffin, mainly because the addition of AGA not only improves the thermal conductivity, but also reduces the relative content of paraffin; thus, the enthalpy is relatively lower. However, the addition of AGA increases the thermal conductivity as well the interaction between AGA and paraffin [46,47], which hinders the movement of the paraffin molecular chain during the phase transition, extending the time to complete the phase transition (it can be seen from Figure 13c that the endothermic peak became gentle). Therefore, the T*_mr_*, T*_fr_*, and T*_c_* of the composites relatively increase as the AGA content increases (i.e., the GO concentration increases). However, in Figure 13d, the T*_mr_* and T*_c_* of PC4W5E15 are lower than those of paraffin, mainly because of its low GO concentration. This leads to the contribution of the interaction between AGA and paraffin to the increase in T*_mr_* and T*_c_*, which is less than the contribution of the increase in thermal conductivity to the decrease in T*_mr_* and T*_c_*. However the T*_fr_* of PC4W5E15 is longer than that of paraffin; this is mainly because the thermal conductivity of solid paraffin is higher than that of liquid paraffin, which makes the T*_sr_* of the paraffin itself shorter than its T*_mr_*. Figure 13e shows the effect of AGAs with the same GO concentration but different microwave treatment time on the paraffin phase transition. It can be seen from the figure that both ∆H*_m_* and ∆H*_f_* of the AGA/paraffin composite materials are lower than those of paraffin, which does not significantly change with the change in microwave treatment time. The main reason is the insignificant change in AGA and relative paraffin contents. The effect of the phase change temperature and phase change process on paraffin phase transition is the same as that shown in Figure 13d. The main influencing factors are the different thermal conductivities of the composites and different interaction forces between AGA and paraffin. The content of groups containing oxygen and nitrogen in AGA treated by microwave for 1 min is more than that treated by microwave for 20 min, which causes a lower thermal conductivity of the composites. However, as can be seen from Figure 3, with the extension of microwave processing time, many fragments and burrs (circle) are observed in the graphene sheets, which results in the lower interaction force between AGA treated by microwave for 1 min and paraffin.

**Figure 14:**
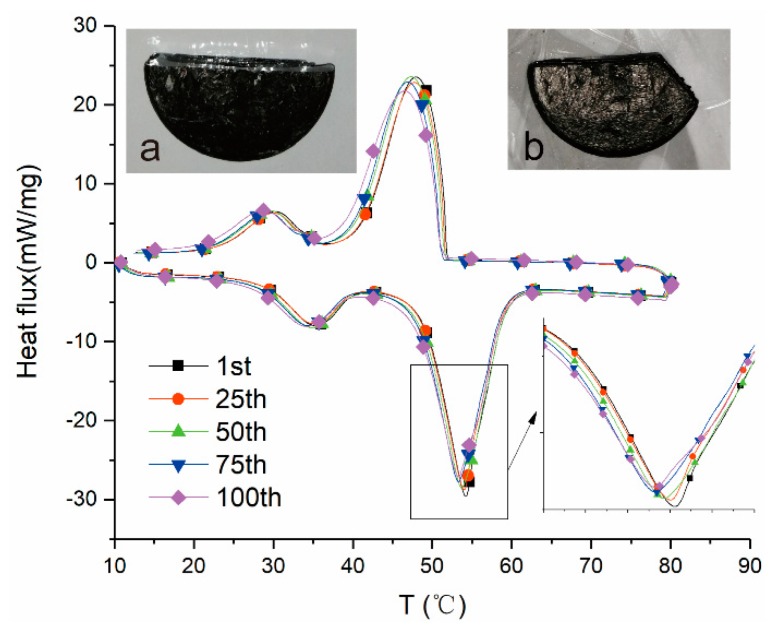
DSC curves of C12W5E15 after several thermal cycles ((**a**,**b**) were digital photos taken before and after 100 cycles respectively).

Thermal cycle reliability and thermal stability are important characteristics of PCMs for thermal energy storage application. Figure 14 presents the thermal cycle test results of the C12W5E15 after 1–100 heating-freezing cycles. The DSC curves of C12W5E15 after 25, 50, 75 and 100 phase change cycles nearly coincide with that of C12W5E15 after the first phase change. After 100 thermal cycles, the latent heat and phase transition temperature of the composite does not change significantly. The initial phase transition temperature difference is within 0.91 °C, and the latent heat decreases by a maximum of 3.2%. Figure 14a,b (The missing part was used for DSC testing) are digital photos taken before and after 100 thermal cycles, respectively. It can be seen that the composite shape remains well, with basically no deformation. Those indicate that the obtained AGA/paraffin composite could keep its phase change behavior after 100 thermal cycles and it has a good thermal cycle stability.

**Figure 15:**
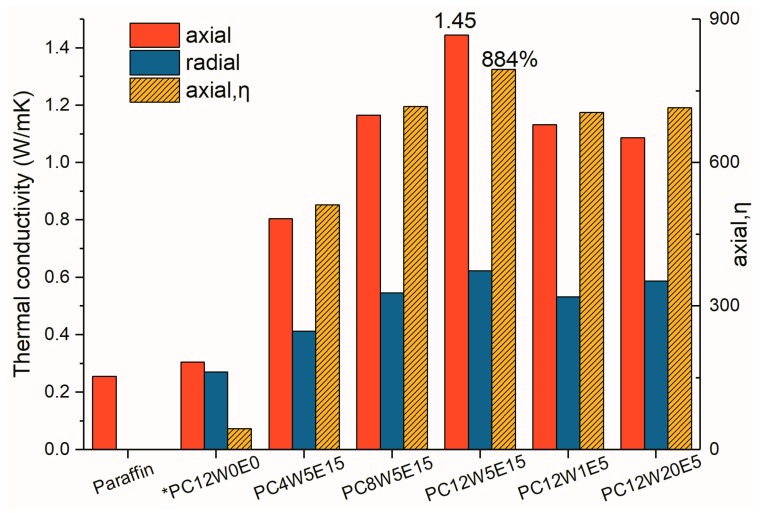
Thermal conductivity and thermal enhancement efficiency of AGA/paraffin composites (where * was prepared by non-heat flow method).

The thermal conductivity of AGA/paraffin composites was studied. Figure 15 shows the thermal conductivity and thermal conductivity enhancement efficiency of the composite. As the AGA content increases (i.e., the GO concentration increased), the thermal conductivity gradually increases; however, the extension of microwave processing time can not increase the thermal conductivity. This is due to the fact that, from XRD and Raman analyses, although microwave treatment can reduce oxygen- and nitrogen-containing groups, it can restore a part of the sp2 domain; however, this treatment produces a large number of fragments and holes, thereby increasing the boundary thermal resistance. The axial thermal conductivity of the composite is significantly higher than the radial thermal conductivity. This is because the axially aligned graphene sheets have a large contact area and a flat heat conduction path, resulting in low thermal resistance and contact thermal resistance. The axial thermal conductivity is 1.46 W/(mK), and the thermal conductivity enhancement efficiency is 844% (the content of AGA is 0.53 vol %). In the radial direction, because the graphene sheets are crosslinked at a certain angle, the heat transfer path becomes tortuous, the contact area between the graphene sheets in the radial direction becomes small, and the boundary graphene sheets become curved [48], thus resulting in higher thermal resistance than that in the axial direction.

**Figure 16:**
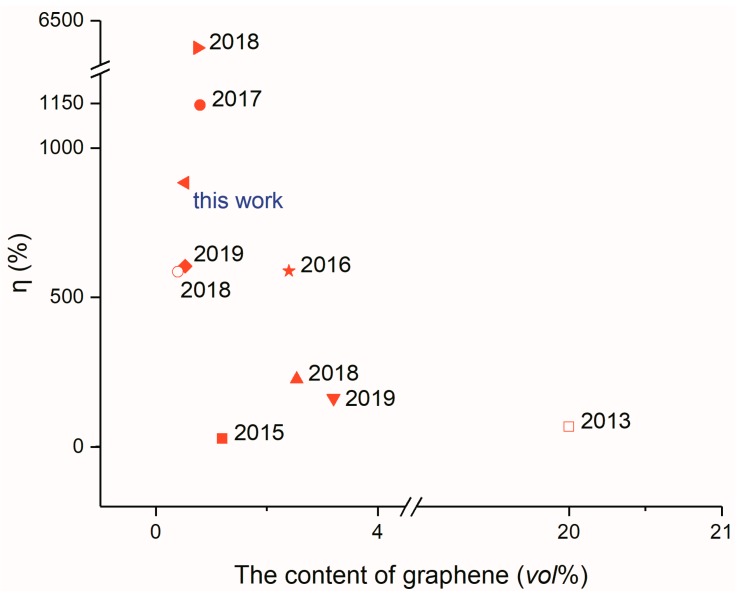
Comparison of thermal conductivity enhancement of graphene/paraffin composites (■ [47] ● [46] ▲ [49] ▼ [50] ► [33] ◆ [51] ★ [34] □ [52] ○ [53]).

In recent years, graphene is extensively reported to enhance the thermal conductivity of composites; however, the effect of thermal conductivity enhancement is considerably different. This is because there are many factors influencing graphene to enhance the thermal conductivity of composites. For example, different preparation methods of graphene will lead to different graphene quality, which will seriously affect the thermal conductivity of graphene itself and ultimately affect the thermal conductivity of those composites. There are different methods of graphene addition, such as direct mixing or preparation of foam or aerogel for adsorption; however, these methods will seriously affect the thermal conduction pathway, thus affecting the thermal conductivity of composites. Moreover, the compatibility of the composites with graphene considerably affects the thermal conductivity of the composites. Figure 16 compares the thermal conductivity enhancement properties of graphene/paraffin composites in recent years. As can be seen from the figure, the AGA/ paraffin composite prepared in this study shows an excellent thermal conductivity enhancement performance, which is better than that reported in most literatures compared, and the AGA consumption is less, but worse than that reported in the [33] and [46]. The main reason for this result is that these two studies carried out high temperature quenching of rGO to repair redox defects. The former involved quenching for two hours under argon protection at a high temperature of 2800 °C, while the latter conducted quenching for 30 min under argon protection at 1000 °C. However, the condition was harsh because of the high-temperature repair and the cost was high because of the protection of argon gas; therefore, it was difficult to produce the composites on a large scale. However, the AGA/paraffin composites prepared in this study have considerable practical significance in production because of mild preparation conditions, low cost and obvious thermal conductivity enhancement efficiency.

**Figure 17:**
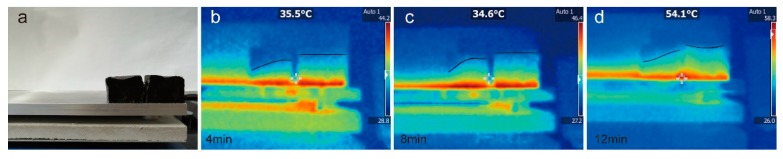
Thermal imaging images of *PC12W0E0 (left) and PC12W5E15 (right). (**a**) was the digital image taken before heating; (**b**), (**c**) and (**d**) were the thermal imaging images taken at 4 min, 8 min and 12 min respectively.

In order to further intuitively evaluate the heat transfer performance of AGA/paraffin composites, an infrared thermal imager was used to study the temperatures of the composites under different heating times. As shown in Figure 17a, the cylindrical sample was cut into two pieces along the diameter of the bottom surface, and one of the two pieces was placed on a constant temperature heating plate at 60 °C for heating; images were obtained with an infrared thermal imager at different times. As can be seen from Figure 17, the temperature (shown in the black curve) of *PC12W0E0 (left) in the entire experiment is lower than that of PC12W5E15 (right). The temperature of *PC12W0E0 increases slowly with the extension of the heating time, but PC12W5E15 quickly transfers heat to the top (the color of the upper half is deepened), thereby reducing the temperature gradient of the entire sample. At 12 min, the PC12W5E15 sample not only increases the temperature of the entire sample, but also shows higher temperatures on both sides, because both sides present an inner ring (Figure 1e), where heat accumulates, while the middle directly transfers heat to the top and is released into the environment. It can be seen from the above experimental results that this composite material can be applied to the PES and heat management medium for rapid storage and release of energy.

## 4. Conclusions

In this study, anisotropic graphene/GO aerogel was prepared by the heat flow method, and high-quality aerogel was obtained by vacuum microwave treatment. After the adsorption of paraffin, its axial thermal conductivity is 1.45 W/(mK) and thermal enhancement efficiency is 884% (AGA content is 0.53 vol %). The TGA and XPS analyses show that after the hydrothermal reduction, most of the oxygen-containing groups that decompose at low temperature (about 200 °C and below) could be reduced and removed, and microwave treatment could further remove a large number of oxygen- and nitrogen-containing groups. However, because of the instantaneous increase in AGA temperature during microwave treatment, a large number of water molecules and oxygen- and nitrogen-containing groups are immediately decomposed and removed, which leads to the expansion and partial stripping of the graphene layer on the pore wall of the aerogels. In addition, as the processing time increases, more oxygen- and nitrogen-containing groups are decomposed and removed, resulting in a large number of holes and that are even broken into many fragments. XRD and Raman analyses show that the diffraction peak of GO completely disappears in the AGAs, and a peak appears around 26.5°; however, the addition of EG leads to a diffraction peak between GO and graphite. DSC analysis shows that the T*_ms_*, ∆H*_m_* and ∆H*_f_* of the AGA/paraffin composite decrease with the increasing GO content in AGA, but the T*_mr_*, T*_fr_* and T_c_ all increase. According to thermal imaging analysis, AGA/paraffin composites show obvious advantages in heat transfer.

In summary, the heat flow method reported in this study has the advantages of simplicity and mild preparation conditions. The prepared anisotropic materials can have broad applications in the fields of PES and thermal energy management.

## Figures and Tables

**Table 1:** Temperature, Density and Enthalpy.

Sample ID	T*_ms_* (°C)	T*_mr_* (°C)	∆H*_m_* (J/g)	T*_fr_* (°C)	∆H*_f_*(J/g)	T*_c_* (°C)	ρ * (mg/cm^3^)
paraffin	48.95	10.69	164.8	10.26	168.9	1.11	/
PC4W5E15	48.81	10.26	161.9	10.56	165.7	0.9	8.53
PC8W5E15	48.80	10.96	159.2	11.81	163.8	2.29	10.06
PC12W5E15	48.75	11.93	153.3	12.24	157.0	2.41	11.87
PC12W1E5	48.81	9.52	159.7	10.86	163.8	0.44	9.87
PC12W20E5	48.74	12.35	159.6	12.34	164.0	1.16	9.23

Note: * AGAs’ density in AGA/Paraffin composites.
